# Inter-rater Reliability of the Qol-measure QUALIDEM

**DOI:** 10.1186/1472-6955-14-S1-S12

**Published:** 2015-10-08

**Authors:** Martin Dichter

**Affiliations:** 1 German Centre for Neurodegenerative Diseases (DZNE), Witten, Germany, and Department of Health, School of Nursing Science, Herdecke University, Witten, Germany

## Background

Improving access to health and nursing care aims to maintain and promote the quality of life (Qol) of care recipients. Especially in dementia research, Qol is a major outcome. QUALIDEM is an often-used dementia-specific Qol measure. It consists of two consecutive versions, one for mild to severe and one for very severe dementia. Previous results showed an insufficient inter-rater reliability (IRR).

## Aims

Development of a user guide for the measurement and the evaluation of the IRR of QUALIDEM.

## Methods

The study was conducted in two steps: (1) A QUALIDEM user guide was developed, based on 11 cognitive interviews with 16 nurses experienced in dementia care. (2) The item “Distribution” and the IRR of the QUALIDEM were evaluated in a field test including n=55 (mild to severe) and n=36 (very severe dementia) residents from nine nursing homes. The people with dementia were assessed four times by blinded proxy-raters. Proxy-raters were nurses and nursing assistants (n=40) who knew the people with dementia well. The proxy-ratings were led by a trained researcher and followed the user guide. The intra-class correlation coefficients (ICC) were calculated for each QUALIDEM subscale separately.

## Results

The user guide includes definitions and examples for each item. The distribution of the responses of all 40 QUALIDEM items (n=40) was examined, indicating floor or ceiling effects for 13 items. All QUALIDEM subscales for people with mild to severe dementia showed a strong IRR (ICC ≥ 0.91). With the exception of one subscale (ICC: 0.64), the version for people with very severe dementia indicated a strong IRR (ICC ≥ 0.79) too. A second analysis excluding the items with floor and ceiling effects confirmed the strong IRR.

## Conclusions

This study provides meaningful evidence for further development of the QUALIDEM and its future use as an outcome measure of dementia care intervention studies.

